# Real-world outcomes of induction chemoimmunotherapy in patients with non–small cell lung cancer who have never smoked

**DOI:** 10.1016/j.xjon.2026.101983

**Published:** 2026-08-03

**Authors:** Alison S. Baskin, Maureen Canavan, Benjamin J. Resio, Daniel J. Boffa, Giorgio Caturegli

**Affiliations:** a Department of Surgery, UCSF, San Francisco, Calif; b Department of General Internal Medicine, COPPER Center, Yale School of Medicine, New Haven, Conn; c Department of Surgery, Yale School of Medicine, New Haven, Conn

**Figure:**
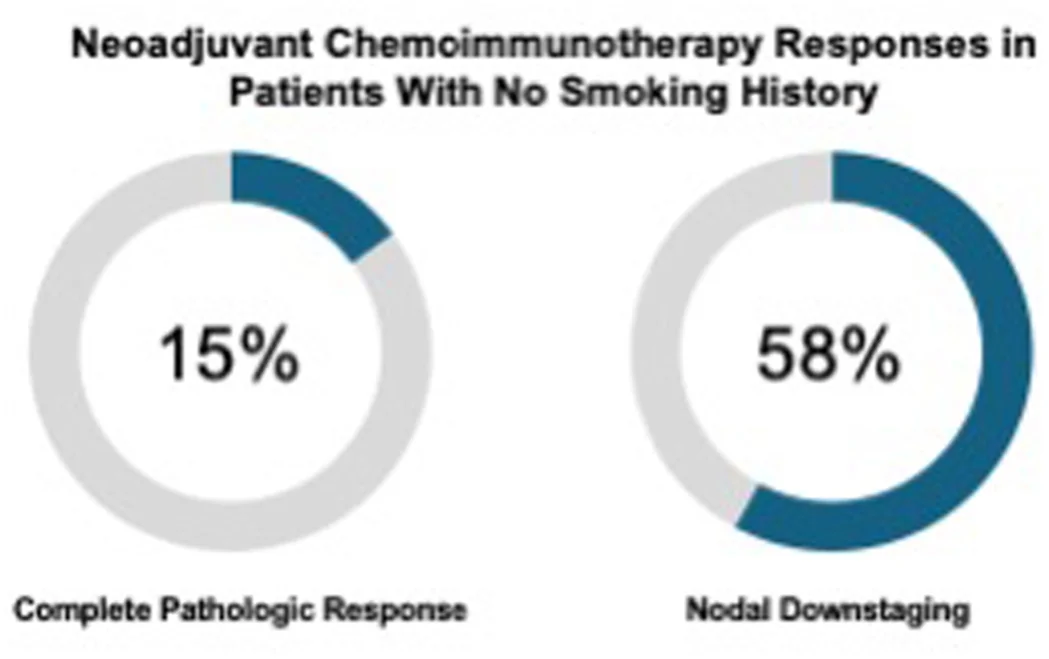
Never smokers showed 15% pCR & 58% nodal downstaging with preoperative chemoimmunotherapy.

**Central Message**

In the largest real-world US study of patients who have never smoked, meaningful responses to induction chemoimmunotherapy were observed, including complete pathologic response and nodal downstaging.

Among patients with non–small cell lung cancer (NSCLC), smoking is associated with tumor and genomic features that may enhance response to chemoimmunotherapy. In contrast, exploratory subgroup analyses from recent clinical trials found that induction chemoimmunotherapy may be less effective among patients without a smoking history, although these investigations were not powered for this subgroup.^1^, ^2^, ^3^, ^4^, ^5^ Despite both the increasing incidence of NSCLC among patients who have never smoked and the growing use of perioperative chemoimmunotherapy, contemporary data describing outcomes within this population remain limited. Using real-world data, we evaluated the impact of neoadjuvant chemoimmunotherapy in patients with no smoking history to explore whether a subset of this population may benefit from induction chemoimmunotherapy.

## Methods

The institutional review board at Yale approved this study exempt with a waiver of informed consent, given deidentified data. The National Cancer Database was queried for adult patients diagnosed in 2023 with clinical stage II-III NSCLC who received neoadjuvant chemoimmunotherapy followed by definitive surgery and had documented smoking status (99.4%). Patients who received neoadjuvant radiation were excluded. Complete pathologic response (pCR) and nodal downstaging (lower pathologic than clinical nodal stage) were evaluated by smoking status (never vs any history of smoking). Univariate analyses were performed to identify independent factors associated with pCR and nodal downstaging among patients with no smoking history.

## Results

Overall, 1532 patients were identified, including 138 (9%) patients with no smoking history and 1394 (91%) with a smoking history (Table E1). A pCR was observed in 15% (n = 21) of patients without and 36% (n = 502) of patients with a smoking history (*P* < .001) (Table 1). Among patients with cN1-3 disease, nodal downstaging occurred in 58% (n = 36/62) of patients without and 75% (n = 621/827) of patients with a smoking history (*P* < .001). Poor/undifferentiated tumor grade was associated with pCR among never smokers (33% vs 7% in well/moderately differentiated tumors, *P* = .03) (Table 2). A similar trend was observed for nodal downstaging, although the association did not reach statistical significance.

**Table 1:** Pathologic outcomes after neoadjuvant chemoimmunotherapy

Outcome	No smoking history (n = 138)	Any smoking history (n = 1394)	*P* value
Complete pathologic response			**<.001**
No	117 (84.8%)	892 (64.0%)	
Yes	21 (15.2%)	502 (36.0%)	
Nodal downstaging (cN1-3, n = 889)			**<.001**
No	26/62 (41.9%)	206/827 (24.9%)	
Yes	36/62 (58.1%)	621/827 (75.1%)	

Bold *P* values represent statistically significant *P* < .05.

**Table 2:** Clinicopathologic factors associated with complete pathologic response (pCR) and nodal downstaging among patients with no smoking history treated with neoadjuvant chemoimmunotherapy

Characteristic∗	pCR† *P* value	Nodal downstaging‡ *P* value
Age	.71	.37
Sex	.71	.34
Race/ethnicity	.84	.43
Region	.12	.55
Urbanicity	.22	.95
Facility type	.16	.95
Insurance status	.55	.65
Median household income	.39	.38
Charlson-Deyo comorbidity index	.51	.11
Clinical T stage	.91	.14
Clinical N stage	.25	.22
Clinical stage	.25	.79
Tumor grade	**.03**	.51
EGFR or ALK mutation	.47	.16
Adenocarcinoma histology	.54	.63
Extent of resection	.30	.92

Bold *P* values represent statistically significant *P* < .05.

∗ Reported in accordance with National Cancer Database reporting rules.

† N = 138.

‡ N = 62.

## Discussion

In this study, representing to our knowledge one of the largest contemporary real-world cohorts of patients with no smoking history treated with neoadjuvant chemoimmunotherapy, 15% achieved a pCR and more than one half of patients who were clinically node-positive experienced nodal downstaging. Although the observed pCR rate was only modestly greater than that reported in CheckMate 816 (15% vs 11%), our substantially larger cohort (138 vs 19 patients) provides real-world evidence that there exists a subset of patients with no smoking history who may derive benefit from immune checkpoint inhibitors.^4^ As expected, patients with no smoking history achieved a pCR and were downstaged less frequently than those with a smoking history. However, the principal contribution of this analysis is not the comparison between smoking groups but the demonstration that clinically meaningful treatment responses can indeed occur in patients who have not smoked, particularly if their tumors are poorly differentiated.

Given the limitations of the National Cancer Database, we were unable to evaluate several factors that may influence chemoimmunotherapy effectiveness (eg, neoadjuvant treatment regimen, tumor mutational burden, programmed death-ligand 1 expression, and other molecular biomarkers). Thus, prospective studies of this population are needed to inform treatment paradigms and determine which patients in this targeted population are most likely to benefit. This investigation will be increasingly important as the proportion of patients with NSCLC who have never smoked continues to increase and systemic therapy options expand. Ultimately, the question may not be whether patients with no smoking history respond to chemoimmunotherapy, but rather, in which cases they do.

## Conflict of Interest Statement

The authors reported no conflicts of interest.

The *Journal* policy requires editors and reviewers to disclose conflicts of interest and to decline handling or reviewing manuscripts for which they may have a conflict of interest. The editors and reviewers of this article have no conflicts of interest.

## Figures and Tables

**Table E1:** Baseline characteristics of patient cohort by smoking history

Patient characteristics	No smoking history (n = 138)	Any smoking history (n = 1394)	*P* value
Age ≥65 y	87 (63.0%)	873 (62.6%)	.92
Female	83 (60.1%)	652 (46.8%)	.003
Race/ethnicity			<.001
Hispanic	12 (8.7%)	61 (4.4%)	
Non-Hispanic White	93 (67.4%)	1134 (81.4%)	
Non-Hispanic Black	<10 (<7.2%)	147 (10.6%)	
American Indian/Alaskan Native	0	<10 (<0.7%)	
Asian	22 (15.9%)	29 (2.1%)	
Native Hawaiian/Pacific Islander	0	<10 (<0.7%)	
Other/unknown	<10 (<7.2%)	17 (1.2%)	
Region			.002
Northeast	≥20 (≥14.5%)	298 (21.4%)	
Midwest	34 (24.6%)	365 (26.1%)	
South	56 (40.6%)	473 (41.1%)	
West	18 (13.0%)	152 (10.9%)	
Unknown	<10 (<7.2%)	106 (7.6%)	
Urbanicity			.08
Metro	≥114 (≥82.6%)	1117 (80.1%)	
Urban	14 (10.1%)	170 (12.2%)	
Rural	0	55 (4.0%)	
Unknown	<10 (<7.2%)	52 (3.7%)	
Facility type			.002
Community	<10 (<7.2%)	61 (4.4%)	
Comprehensive community	45 (32.6%)	458 (32.1%)	
Academic/research	58 (42.0%)	≥595 (≥42.7%)	
Integrated network	23 (16.7%)	280 (20.1%)	
Unknown	<10 (<7.2%)	<10 (<0.7%)	
Insurance status			.02
Uninsured	<10 (<7.2%)	13 (0.9%)	
Private	47 (34.1%)	391 (28.1%)	
Medicaid	<10 (<7.2%)	111 (8.0%)	
Medicare	78 (56.5%)	832 (59.7%)	
Other	<10 (<7.2%)	37 (2.7%)	
Unknown	<10 (<7.2%)	10 (0.7%)	
Median income, $			.28
<46,277	10 (7.3%)	177 (12.7%)	
46,277-57,856	19 (13.8%)	223 (16.0%)	
57,857-74,062	33 (23.9%)	276 (19.8%)	
>74,062	43 (31.2%)	387 (27.8%)	
Unknown	33 (23.9%)	331 (23.7%)	
Charlson-Deyo			<.001
0	107 (77.5%)	799 (57.3%)	
1	23 (16.7%)	337 (24.2%)	
2	<10 (<7.2%)	156 (11.2%)	
≥3	<10 (<7.2%)	102 (7.3%)	
Clinical T			.003
1	15 (10.9%)	326 (23.4%)	
2	37 (26.8%)	376 (27.0%)	
3	51 (37.0%)	445 (31.9%)	
4	35 (25.4%)	247 (17.7%)	
Clinical N			.002
0	≥68 (≥49.3%)	567 (40.7%)	
1	18 (13.0%)	361 (25.9%)	
2	42 (30.4%)	455 (32.6%)	
3	<10 (<7.2%)	11 (0.8%)	
Clinical stage			.99
2	62 (44.9%)	626 (44.9%)	
3	76 (55.1%)	768 (55.1%)	
Clinical tumor grade			.50
Well/moderate	27 (19.6%)	220 (15.8%)	
Poor/undifferentiated	21 (15.2%)	232 (16.6%)	
Unknown	90 (65.2%)	942 (67.6%)	
Adenocarcinoma	87 (63.0%)	692 (49.6%)	.003
EGFR or ALK mutation	18 (13.0%)	46 (3.3%)	<.001
Extent of resection			.26
Wedge resection or segmentectomy	<10 (<7.2%)	68 (4.9%)	
Lobectomy	126 (91.3%)	1258 (90.2%)	
Pneumonectomy	<10 (<7.2%)	68 (4.9%)	
